# Cleidocranial dysplasia in a Moroccan patient: a case report

**DOI:** 10.11604/pamj.2026.53.51.48261

**Published:** 2026-02-03

**Authors:** Hafsa Mansy, Khadija Oumensour, Jamila Ait Ikiss, Samira Elarabi

**Affiliations:** 1 Department of Orthodontics and Dentofacial Orthopedics, Faculty of Dentistry, Hassan II University, Casablanca, Morocco,; 2 Department of Pediatric Dentistry-Prevention, Faculty of Dentistry, Hassan II University, Casablanca, Morocco

**Keywords:** Rare disease, diagnosis, role of the pediatric dentist, cleidocranial dysostosis, case report

## Abstract

Cleidocranial dysplasia is a rare autosomal dominant disorder characterized by skeletal and dental anomalies, often enabling early recognition by dental practitioners. A 14-year-old Moroccan girl presented to the Pediatric Dentistry Department of Ibn Rochd University Hospital with aesthetic concerns. Clinical and radiographic examinations revealed multiple general, craniofacial, and dental abnormalities that suggested an underlying syndrome. Further investigations supported the diagnosis of cleidocranial dysplasia. The patient received appropriate dental care and was referred to the genetics department for specialized follow-up, along with a detailed report summarizing the oro-dental findings. This case highlights the essential role of pediatric dentists in identifying syndromic patterns and recognizing rare diseases when multiple anomalies coexist.

## Introduction

A rare disease is a disabling condition affecting one in 2,000 people according to the European Organization for Rare Diseases (EURODIS) [1,2]. While this prevalence is commonly reported across several countries, some nations, such as Denmark, Sweden, and the United Kingdom, report even lower prevalence rates, ranging between 1 in 10,000 and 1 in 50,000 [3]. To date, an estimated 6,000 to 8,000 rare diseases have been identified, with over one and a half million individuals affected in Morocco alone [3]. Most rare diseases are of genetic origin [2-4], with approximately 70 to 80% classified as such, according to the National Institutes of Health (NIH) [5]. However, the etiology of a significant number of these diseases (around 20 to 30%) remains unknown [3,5]. Due to the limited number of cases, the scarcity of knowledge and information, and the fact that certain pathognomonic manifestations may only appear at a later stage, the diagnosis of rare diseases is often delayed and challenging [2-4].

Rare diseases represent a real public health challenge [4], given their number, rising incidence, clinical severity, and, in most cases, the absence of etiological treatment. Effective management requires the involvement and collaboration of various medical specialties, such as pediatrics and medical genetics, to accelerate the diagnostic process. Dentists, for their part, play an important role in the diagnosis, referral, and management of rare diseases [3]. These conditions often manifest with a wide range of clinical signs, especially in the oro-facial region. According to current literature, 900 rare diseases involve dento-oro-facial manifestations, and 750 are associated with oro-facial clefts [2,3]. We report the case of a 14-year-old patient whose constellation of dental, craniofacial, and general anomalies observed during clinical and radiographic examinations allowed us to establish the diagnosis of cleidocranial dysostosis, which was subsequently confirmed by the Medical Genetics and Molecular Biology Department of Ibn Rochd University Hospital.

## Patient and observation

**Patient information:** a 14-year-old female patient, M.A., presented to the Pediatric Dentistry Department of Ibn Rochd University Hospital for oral cavity rehabilitation. She had no notable medical or surgical history.

**Figure 1:**
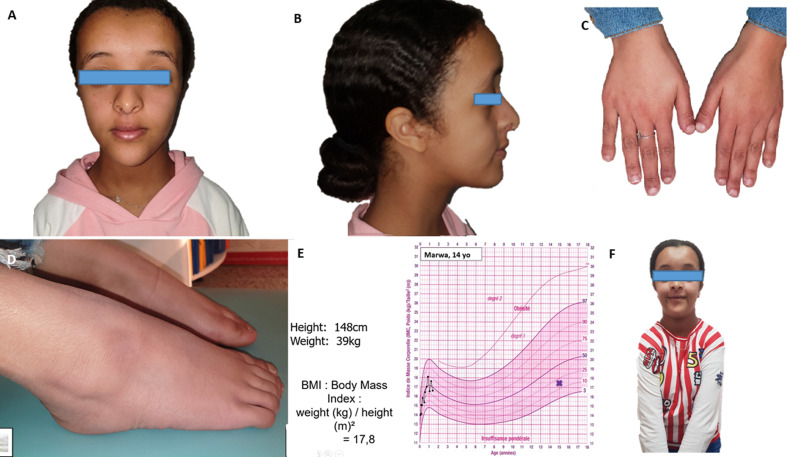
extraoral clinical features of the patient: A) frontal facial view; B) profile view; C) examination of the hands showing dysmorphic features; D) examination of the feet showing dysmorphic features; E) patient´s position on the girls´ growth chart; F) frontal view of the thoracic region showing shoulder hypermobility

**Clinical findings:** a general and dysmorphic examination was performed (Figure 1A-F), revealing several anomalies, including: persistent open cranial sutures, a triangular-shaped face, midface prominence, prominent frontal bosses, hypertelorism, a depressed nasal bridge, flattened cheekbones, nasal septum deviation, a long philtrum, a pointed chin, and drooping shoulders. In addition, staturo-ponderal insufficiency was noted and confirmed by plotting the patient´s growth on a standard growth chart.

Examination of the hands and feet revealed hypoplastic distal and middle phalanges, short and broad thumbs, and flat feet with an absent plantar arch (Figure 1C, D). Furthermore, a particularly notable feature was clavicular hypoplasia, which allowed for abnormal mobility of the clavicles (Figure 1F).

**Figure 2:**
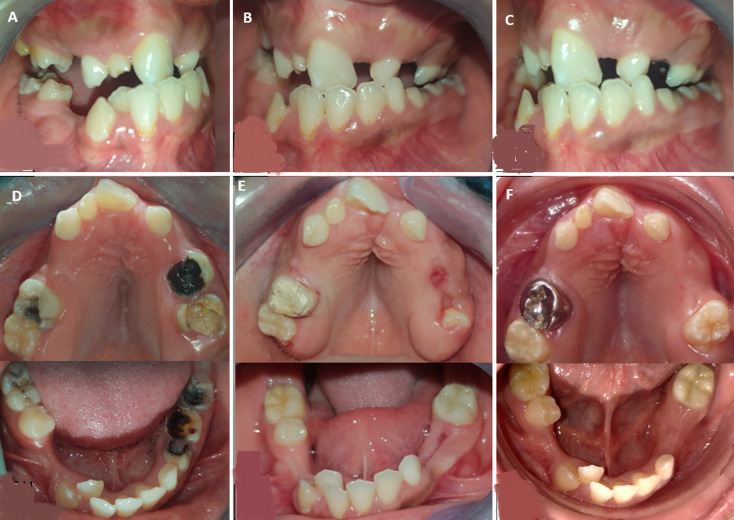
intraoral clinical findings at different stages: A) right lateral intraoral view before treatment; B) frontal intraoral view before treatment; C) left lateral intraoral view before treatment; D) occlusal views of the maxilla and mandible before treatment; E) occlusal views of the maxilla and mandible after treatment; F) occlusal views of the maxilla and mandible three years after treatment

Assessment of oral functions showed mixed breathing, atypical swallowing, and impaired phonation of the phonemes “R” and “L”, accompanied by nasality. Intraoral examination (Figure 2A-F) revealed poor oral hygiene, persistence of deciduous teeth, clinical absence of several permanent teeth, multiple carious lesions, and a disrupted occlusal environment. Additionally, a short lingual frenulum was noted.

**Timeline of current episode:** the patient is the offspring of a first-degree consanguineous marriage. Her psychomotor and cognitive development was reported as satisfactory. However, the mother mentioned that a craniofacial malformation had previously been noted by a general practitioner, although no specific diagnosis or further details were provided.

**Figure 3:**
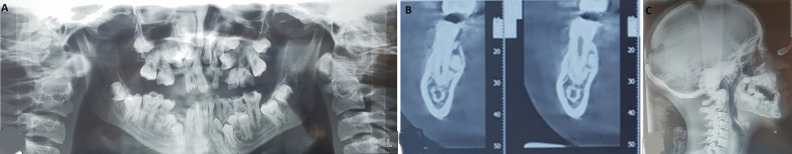
radiographic examinations of the patient: A) panoramic radiograph; B) oblique cross-sectional images from cone beam computed tomography (CBCT) showing an additional unerupted tooth germ located lingually to the right mandibular second premolar (#45); C) lateral cephalometric radiograph

**Diagnostic assessment:** firstly, we requested a panoramic radiograph (Figure 3A). This examination provided an initial overview of the patient´s dental development, confirming the presence of unerupted permanent teeth, including premolars, second permanent molars, and wisdom teeth. Three additional tooth germs were also identified on the lower left side, and one on the lower right. A fourth and final supernumerary tooth was located lingual to the right lower second premolar (#45), as visualized using cone beam computed tomography (CBCT) (Figure 3B). To confirm the presence of disharmonious maxillo-mandibular development, a lateral cephalometric radiograph was prescribed. This revealed an abnormally radiolucent appearance of the cranial vault sutures, corroborating the clinical finding of open cranial sutures on palpation (Figure 3C).

The presence of general and cranio-facial dysmorphism associated with all the dental manifestations exposed led us to suspect the presence of a syndromic disease. To investigate further, we explored both general and dental manifestations using two online databases: OMIM and Orphanet. Several diagnostic hypotheses emerged from this search, the most relevant of which was Cleidocranial Dysostosis (CCD). Simultaneously, the patient was referred to the Medical Genetics Department with this provisional diagnosis and a detailed report of the bucco-dental anomalies for further evaluation. During the initial consultation, a series of complementary examinations was prescribed, including frontal chest, hand and wrist, pelvis, and knee radiographs. These investigations served to complete the clinical profile of the patient. Following this evaluation and the interpretation of the radiographic findings, the medical genetics team provided a preliminary confirmation of our diagnostic hypothesis.

**Diagnosis:** all the examinations carried out allowed us to retain the following diagnoses: skeletal class III associated with maxillary endognathia; deep amelo-dentinal lesions on the six-year molars, deep lesions with pulpal involvement on the persistent deciduous molars; multiple dental anomalies: inclusion of the following permanent teeth 17, 15, 14, 13, 12, 21, 22, 23, 24, 25, 27, 37, 35, 34, 33, 44 and 47, and the presence of 4 lower supernumerary germs: 2 right premolar germs, one left canine germ, and 2 left premolar germs.

**Therapeutic interventions:** a two-phase treatment plan was proposed for the patient: the first phase focused on the rehabilitation of the oral cavity, with dental care performed by quadrant to save time and enhance efficiency. Given the limited number of teeth present in the arch, our therapeutic approach prioritized conservation over extractions. Following oral hygiene education and motivation, the treatments carried out were as follows: dentino-pulpal therapies combined with composite restorations on teeth 46 and 36; extractions of teeth 74, 75, 65, and 26; a preventive pulpotomy on tooth 55, which was then restored using a preformed pediatric crown; and pit and fissure sealants applied to teeth 16 and 45 due to the patient´s high caries risk.

After interdisciplinary consultation with our orthodontist and periodontologist colleagues at the Ibn Rochd University Hospital, a second, orthodontic-surgical phase was planned to achieve both functional and aesthetic occlusion. The first step in this phase was a lingual frenectomy. A combined orthodontic-surgical treatment was proposed, including the surgical extraction of retained deciduous teeth and supernumerary tooth germs impeding spontaneous eruption and orthodontic mechanics. This would be followed by orthognathic surgery to correct the severe skeletal dysmorphism, along with orthodontic traction of impacted permanent teeth. However, because of financial constraints, the patient and her guardian decided to delay this phase of treatment.

**Follow-up and outcome of interventions:** the patient was followed up for three years to monitor the outcomes of the initial care and to maintain motivation for proper oral hygiene (Figure 2F).

**Patient perspective:** now aware of her rare disease, the patient and her guardian showed motivation during and after the first phase of treatment and expressed their intention to continue the treatment as soon as their financial situation allows.

**Informed consent:** given the rare nature of her condition, written informed consent was obtained and signed by her legal guardian in the patient´s presence. The form authorizes the publication of personal data, including facial, profile, and intraoral photographs, for scientific purposes.

## Discussion

The clinical and radiographic examinations revealed syndromic dental anomalies, supported by the presence of both oral and systemic signs. The concurrence of several dental anomalies: multiple impactions, delayed eruption of permanent teeth, persistence of deciduous teeth, and the presence of supernumerary germs, prompted suspicion of a syndromic condition.

To establish a diagnostic hypothesis, we compiled the most significant findings and searched for them using the open-access databases OMIM and Orphanet. OMIM generated several syndromic possibilities, classified by relevance, with Cleidocranial Dysostosis (CCD, #119600) ranking as the most consistent match. Also known as cleidocranial dysostosis, osteodentin dysplasia, mutational dysostosis, or Marie and Sainton disease, cleidocranial dysostosis is a rare condition characterized by a range of skeletal and dental abnormalities. It was first described in 1898 by Marie *et al*. under the term “hereditary cleidocranial dysostosis” [6].

Cleidocranial Dysostosis (CCD) affects males and females equally, with an estimated prevalence of 0.5 per 100,000 live births, according to ORPHANET and EURODIS [1]. Furthermore, children are most often diagnosed between 10 and 15 years of age, as was the case for our patient. This medical condition follows an autosomal dominant inheritance pattern, although approximately 40% of cases are due to de novo mutations (sporadic mutations), with nearly one-third (33%) of affected individuals having unaffected parents [7].

Cleidocranial Dysostosis (CCD) is caused by a defect in the CBFA1 gene (the core binding factor a-1), also known as RUNX2, and located on the short arm of chromosome 6p21. This gene is critical for normal bone and dental development. In fact, RUNX2 regulates gene expression in mesenchymal cells of the dental epithelium. Mutations lead to impaired ossification of endochondral and membranous bones in the craniofacial region, clavicles, teeth, and pelvis [7]. The delayed dental eruption and tooth impactions observed in CCD patients are partly explained by a reduced number of osteoclasts. A study by Yoda *et al*. [8] on heterozygous Cbfa1+/- mice demonstrated decreased osteoclast numbers, which impairs the physiological resorption of alveolar bone during tooth eruption.

While the disease can affect various bones, the classic diagnostic triad includes craniofacial anomalies, clavicular hypoplasia or aplasia, and pelvic malformations, often accompanied by staturo-ponderal delay. Clinical severity varies widely, ranging from mild to severe phenotype with functional repercussions. The clinical and radiological manifestations associated with this disease could be grouped by areas [9]: 1) skull: brachycephaly, persistent anterior fontanelle, open cranial sutures, and multiple Wormian bones; 2) jaws: maxillary hypoplasia, delayed eruption or impaction of permanent teeth, persistence of deciduous teeth, and multiple impacted supernumerary teeth; 3) thorax: hypoplastic or aplastic clavicles, small bell-shaped thorax with oblique and short ribs; 4) hands: short, tapered fingers, phalangeal anomalies, and malformations of carpal and metacarpal bones; 5) pelvis: widened pubic symphysis, hypoplastic iliac wings, enlarged sacroiliac joints, and coxa vara due to broad femoral necks.

Given the variability of symptoms, a standardized therapeutic strategy is difficult to establish. Management must be individualized, taking into account patient-specific factors such as age, motivation, financial means, and the severity of the condition. Therapeutic management of CCD is challenging. There is no curative treatment for the bone anomalies [10]. However, various bucco-dental interventions are available to improve both function and aesthetics, which is essential for the psychosocial development of affected children [10].

For our patient, ortho-surgical treatment was the ideal therapeutic option. This approach avoids early prosthetic solutions and multiple extractions. It includes the surgical removal of obstacles (non-exfoliated deciduous teeth, supernumerary germs, and alveolar bone) to allow for orthodontic traction of impacted teeth. According to the current data, there are four main approaches to managing the oral manifestations of CCD, as detailed in Table 1 [9,10].

**Table 1:** four approaches for the management of bucco-dental manifestations of cleidocranial dysostosis

Age-specific strategies	
**Approach**	**Therapeutic strategy**
Toronto-Melbourne: multiple surgery approach	5-6 years: extraction of deciduous incisors; 6-7 years: surgical exposure of permanent incisors + bracket bonding; 9-12 years: extraction of deciduous molars; surgical exposure of permanent canines and premolars + bracket bonding; extraction of supernumerary teeth
Jerusalem: two-stage surgical approach	Phase 1: 10-12 years: extraction of anterior deciduous teeth; extraction of all supernumerary teeth; surgical exposure of permanent incisors + bracket bonding. Phase 2: 13 years and older: extraction of deciduous molars; surgical exposure of permanent canines and premolars + bracket bonding
**Age-nonspecific strategies**	
**Approach**	**Therapeutic strategy**
Bronx: surgical-prosthetic approach	First intervention: extraction of supernumerary germs and deciduous teeth. Second intervention: surgical exposure of impacted teeth + bracket bonding; temporization with a removable prosthesis. Third intervention: orthognathic surgery (if necessary)
Belfast-Hambourg: single surgical intervention approach	One intervention: extraction of DT and supernumerary teeth, surgical exposure of permanent teeth + bracket bonding when accessible

Early diagnosis significantly improves prognosis, especially when patients are young and motivated. The overall outlook is also influenced by the quality of care and the potential for complications, including scoliosis, osteoporosis, recurrent respiratory infections, sleep apnea, motor delay, and varying degrees of hearing loss.

The presence of multiple dental anomalies alongside systemic signs should alert clinicians, especially pediatric dentists, to the possibility of a rare genetic disorder [2,3]. Early detection and referral can significantly reduce diagnostic delays and improve patient outcomes. Thus, pediatric dentists play a crucial role in recognizing syndromic patterns, establishing an oral assessment, and facilitating prompt referral to medical genetics for confirmation.

## Conclusion

This case involves a teenage girl diagnosed with cleidocranial dysostosis following clinical, radiographic, and genetic assessment. It illustrates how a careful dental examination can reveal key syndromic features that may otherwise remain unnoticed, underscoring the essential role of pediatric dentists in initiating the diagnostic pathway for rare disorders. Recognizing the characteristic dental and craniofacial pattern of cleidocranial dysostosis enables earlier referral, timely confirmation, and more appropriate multidisciplinary management, ultimately improving long-term prognosis and quality of life.
